# Epidemilogical Profile of Speech and Language Disorder in North Central Nigeria

**Published:** 2011-12

**Authors:** Shuaib K. Aremu, Olushola A. Afolabi, Biodun S. Alabi, Isiah O. Elemunkan

**Affiliations:** *University of Ilorin Teaching Hospital, Ilorin, Nigeria*

**Keywords:** speech disorder, language disorder, deaf-mutism

## Abstract

**Background:**

Speech-language pathologists/Otolaryngologists recognize high prevalence of speech and language disorder among children. The aim of the study is to find out the epidemiological profile of speech and language disorder in north central Nigeria.

**Method:**

A five year retrospective review of all referral to speech and language therapy unit ENT Department between January 2005 and December 2009. Information retrieved and analysed included bio-data, clinical presentation and diagnosis of the patients. Descriptive analysis of these data were done.

**Results:**

A total of 146 patients were seen out of which 89 (61%) were under five, 32 (21.9%) were between 5-10 years, 20 (13.7%) in the range of 11-20 yrs and 2 (1.4%) were between 21-34 yrs. None was observed in the elderly. Male preponderance was noted with male to female ratio of 1.9:1.0. The commonest diagnosis was deaf-mutism in 84 (57.5%) followed by delayed speech development in 31(21.2%) patients. The least diagnosis was aphasia in 2 (1.4%) patients.

**Conclusion:**

Speech and language disorder was commonest among the under five’s with non in the elderly, There was a higher prevalence amongst males and deaf-mutism was the commonest observed.

## INTRODUCTION

Speech-language pathologist/Otorhinolaryngologist recognizes that substantial segments of the adult population as well as younger groups have serious speech, language, and voice defects. Some researchers have reported prevalence as high as 10% for some groups of children (1). Most of these difficulties in children involve functional articulation disorders; faulty language formulation and dysfluency (stuttering) comprise the remainder. In addition, many neurogenic speech and language disorders, unlike stuttering and many articulatory disorders, develop in adulthood (1).

Speech and language disorders can be divided into three major categories. The first and most common are the articulation disorders (2). These problems involve the production of defective speech sounds and sound combinations that may be distorted, omitted, substituted, or added as accessory sounds. Sometimes articulatory disorders are the result of neurogenic disorders and dysarthrias (2). A second type of disorder is the impairment of speech fluency, called stuttering or stammering. Repetition of sounds, syllables, words, or phrases; sound prolongations; atypical pauses (hesitations); word substitution; and use of word fillers characterize dysfluent behavior. A third category is variously labeled language impairment, linguistic disability, or faulty symbolization, which refers to disorders in both expression of thought through verbal language and its comprehension. This category includes various disorders, ranging from delayed/deviant language development to neurogenic disorders known as aphasia (2). The normal acquisition of speech depends on the process of maturation. A “speech readiness period” extends from birth to the fifth year of life, when the child acquires the ability to develop speech as a method of communication which represents the cumulative learning of many bits and pieces of language that begin within the first few weeks of life (3). In the first few months of life, infants are capable of perceptually differentiating a wide variety of speech sound contrasts, giving children the capability of learning any of the languages of the world (4, 5). But, by 6 months of age, children have already begun to lose some of the capability of perceptually differentiating among sound contrasts that are not used by talkers within their immediate language environment (6). Also, by 6 months of age, infants show an enhanced ability to distinguish perceptually those sounds used by talkers within their native language environment (7). Infants and young children are typically “bathed in language” by their caregivers during the first months of life. Such stimulation, often called mothers, serves to nurture proper language development (8). This study aims to find out the epidemiological profile of speech and language disorder in north central Nigeria as there is paucity of data on this subject in this part of the world.

## METHODOLOGY

This was a retrospective study of all patient referred to the speech therapy unit of the department of Ear, Nose and Throat of the university of Ilorin teaching hospital over a five year period through January 2005 to December 2009. Using the patients’ registers and individual case-notes, the data retrieved included the age of the patients, the sex, occupation and the diagnosis. All these were entered into an SPSS version 11.0 computer software and analysed descriptively.

## RESULTS

One hundred and fifty one patients were found to have one form of speech pathology or the other. However five patients were excluded on the basis of inadequate data or misplaced record at the health records department of the hospital, thus one hundred and forty-six patients in these form the basis for this write up. Age grouping showed that 89 (61%) were under five, 32 (21.9%) were between 5-10 years, 20 (13.7%) in the range of 11-20 yrs and non was observed in the elderly (Table 1). There was male preponderance with 96 males and 50 females (Table 3); This gives a male to female ratio of 1.9:1.0.More than half 84 (57.5%) of the patients had a diagnosis of deaf-mutism, about one third 31 (21.2%) had delayed speech development. 6 (4.1%) had slurred speech pathology, 19 (13.0%) had impaired speech, 4 (2.7%) had stammering or stuttering as the diagnosis and only 2 (1.4%) had a aphasia (Table 2). Most of the diagnosis made were commoner in males more than the females except in slurred speech where there is equal prevalence in both sexes and aphasia where females are more prevalent. The diagnosis of deaf mutism was most common among the under fives as seen in (Table 4) below and almost non existent above fifteen years age groups. This was found to apply to all the problems of speech and language disorders, except for slurred speech noticed in two patients in the fourth to fifth decades of life.

**Table 1:** Age Distribution

Age	Frequency	Percentage (%)

0-5 yrs	89	61
6-10 yrs	32	21.9
11-20 yrs	20	13.7
21-34 yrs	2	1.4
35-64 yrs	3	2.0
Total	146	100

**Table 2:** Diagnosis

Diagnosis	Frequency	Percentage (%)

Deaf-Mutism	84	57.5
Delayed speech	31	21.2
Slurred speech	6	4.1
Impaired speech	19	13.0
Stuttering/Stammering	4	2.7
Aphasia	2	1.4
Total	146	100

**Table 3:** Relationship of Sex with diagnosis of patient

Diagnosis	Deaf-Mutism	Delayed	Slurred	Impaired	Stuttered	Aphais	Total

Sex							
Male	58	21	3	11	3	0	96
Female	26	10	3	8	1	2	50
Total	84	31	6	19	4	2	146

**Table 4:** Age of patients with diagnosis of patient

Age group	Diagnosis						Total
	Deaf-mutism	Delay	Slurr	Impair	Stutt	Aphas	

0-5 yrs	51	20	3	13	1	1	89
6-10 yrs	18	8	1	4	-	1	32
11-15 yrs	12	3	-	1	2	-	18
16-20 yrs	1	-	-	-	-	-	1
21-25 yrs	-	-	-	-	-	-	0
26-30 yrs	1	-	-	-	1	-	2
31-35 yrs	-	-	-	-	-	-	0
36-40 yrs	1	-	-	1	-	-	2
41-45 yrs	-	-	-	-	-	-	0
46-50 yrs	-	-	2	-	-	-	2
Total	84	31	6	19	4	2	146

## DISCUSSION

Prevalence of communication disorders in children is high, with a gradual decline through adolescence. At least 5% of school-age children (up to age 17) have significant speech impairment, although most professionals consider this estimate extremely conservative (1). Some researchers have reported prevalence as high as 10% for certain samples of children (1). However from our study the prevalence was found to be 8.6%. The majority of referrals were between 3 months and 5 years old with more boys than girls. The socio-economic status matched that of the local population, similar to findings by Broomfield and Dodd (9). This could be associated with the significant improvements in context of normal speech development, accuracy of production of words of increasing length with increasing age (10).

Stuttering is used to describe a child’s speech when it is marked by effortless repetitions or prolongations of words, phrases, or syllables without awareness on the child’s part that these mannerisms are different or abnormal. This can be primary or secondary (2), Johnson stated that “practically all stutterers are originally diagnosed (regarded as “stutterers,” “not talking right,” or “having difficulty saying words,” and so forth) by their parents, more often than not the mother being the first to become concerned (11).” From our studies only three were found and it constituted 2.7% of speech disorders, however when parents have had a background of experience with stuttering, they appear to react to the speech imperfections of their children differently.

Our studies have shown that male stutterers outnumber female stutterers by about 3 to 1 similar to finding in the literature and this could be from physical standpoint (2). According to Shames, the family is a critical factor in dealing with the young child stuttering because “the family can either reinforce or counteract the efforts of the speech-language pathologist (12).” An advanced, or secondary, stutterer should seek the assistance of a qualified speech-language pathologist. Clinical management of stuttering involves changing the stutterer’s attitude, method of talking, and/or environment. Delayed language development is common and has serious sequelae into adulthood in terms of educational, social and emotional development (13).

From our report there is high percentage of deaf-mutism constituting more than half of the patients seen and this is found to be commoner among male more than the female this is not surprising as the study was conducted in Ilorin which is one of the major Islamic town and also belongs to the northern part of the country and favours consanguineous marriage which has been implicated as a risk factor in the development of deaf-mutism from previous studies (14-16).

Deaf-Mutism is defines as congenital deafness that results in inability to speak (17). This is usually a childhood problem however from our studies some patient present at adult thus the prognosis for this is poor compared to those detected early and started on speech training. The basis of mutism is usually family psychopathology. Mutism most especially in this group could be an elective type in which there is willingness to speak in a limited number of situations but refusal to speak in others (18). A substantial minority of children with elective mutism have a history of speech delay or articulation problems. It is common for elective mutism to be associated with social anxiety, excessive sensitivity to the reactions of others and stubbornness based on fearfulness (14-16).

Elective mutism most frequently appears in early childhood and occurs with approximately the same frequency in both sexes (14) while some found it to be commoner among females than males (18), however this is at variance with our study that found more males than females (14-16).

Delayed language may be mild or severe, once the child's language does appear, it usually develops normally in sequence and pattern (13, 19). Whilst early delay may resolve itself, it may turn out to be a long-term delay or disorder (19). It is the second commonest disorder from our series the degree of which was not determined by the study it may probably be associated with hearing impairment. Some studies have shown that children with conductive hearing loss associated with middle ear fluid during the first few years of life are at risk for speech delay (20, 21). However, not all studies find this association (22). This was not established by our study. The condition is more common in boys, and a family history of "late bloomers" is often present from previous studies (23).

The prognosis for these children is excellent, however; study have shown that they usually have normal speech development by the age of school entry (24).

Limitation of this study is that it is a retrospective study thus the inability to assess the degree of mutism, the relationship between hearing loss and speech and language disorders also the relationship between consanguinity and deaf mutism. Future studies will want to assess relationship between their academic performance and speech and language disorders.

## CONCLUSION

Deaf-mutism is still the commonest speech disorder in our environment common among males more than females and the least common is aphasia, thus the need early hearing screening for early detection of speech disorders and rehabilitation of this group of patients as early detection and rehabilitation gives a better prognosis. Although this study does not find out the nature of marriages but observed a high rate of consanguineous marriages in the community. This is a risk factor to development of speech and language disorders from previous studies and literatures and the need to discourage it so as to reduce the prevalence of speech and language disorders in our environments.
